# Identification and validation of a novel cuproptosis-related gene signature in multiple myeloma

**DOI:** 10.3389/fcell.2023.1159355

**Published:** 2023-04-20

**Authors:** Bingxin Zhang, Quanqiang Wang, Tianyu Zhang, Ziwei Zheng, Zhili Lin, Shujuan Zhou, Dong Zheng, Zixing Chen, Sisi Zheng, Yu Zhang, Xuanru Lin, Rujiao Dong, Jingjing Chen, Honglan Qian, Xudong Hu, Yan Zhuang, Qianying Zhang, Zhouxiang Jin, Songfu Jiang, Yongyong Ma

**Affiliations:** ^1^ Department of Hematology, The First Affiliated Hospital of Wenzhou Medical University, Wenzhou, Zhejiang, China; ^2^ Department of Hepatobiliary Surgery, The Second Affiliated Hospital and Yuying Children’s Hospital of Wenzhou Medical University, Wenzhou, Zhejiang, China; ^3^ Key Laboratory of Intelligent Treatment and Life Support for Critical Diseases of Zhejiang Province, Wenzhou, Zhejiang, China; ^4^ Zhejiang Engineering Research Center for Hospital Emergency and Process Digitization, Wenzhou, Zhejiang, China

**Keywords:** multiple myeloma, cuproptosis, prognostic gene signature, tumor microenvironment, tricarboxylic acid cycle

## Abstract

**Background:** Cuproptosis is a newly identified unique copper-triggered modality of mitochondrial cell death, distinct from known death mechanisms such as necroptosis, pyroptosis, and ferroptosis. Multiple myeloma (MM) is a hematologic neoplasm characterized by the malignant proliferation of plasma cells. In the development of MM, almost all patients undergo a relatively benign course from monoclonal gammopathy of undetermined significance (MGUS) to smoldering myeloma (SMM), which further progresses to active myeloma. However, the prognostic value of cuproptosis in MM remains unknown.

**Method:** In this study, we systematically investigated the genetic variants, expression patterns, and prognostic value of cuproptosis-related genes (CRGs) in MM. CRG scores derived from the prognostic model were used to perform the risk stratification of MM patients. We then explored their differences in clinical characteristics and immune patterns and assessed their value in prognosis prediction and treatment response. Nomograms were also developed to improve predictive accuracy and clinical applicability. Finally, we collected MM cell lines and patient samples to validate marker gene expression by quantitative real-time PCR (qRT-PCR).

**Results:** The evolution from MGUS and SMM to MM was also accompanied by differences in the CRG expression profile. Then, a well-performing cuproptosis-related risk model was developed to predict prognosis in MM and was validated in two external cohorts. The high-risk group exhibited higher clinical risk indicators. Cox regression analyses showed that the model was an independent prognostic predictor in MM. Patients in the high-risk group had significantly lower survival rates than those in the low-risk group (*p* < 0.001). Meanwhile, CRG scores were significantly correlated with immune infiltration, stemness index and immunotherapy sensitivity. We further revealed the close association between CRG scores and mitochondrial metabolism. Subsequently, the prediction nomogram showed good predictive power and calibration. Finally, the prognostic CRGs were further validated by qRT-PCR *in vitro*.

**Conclusion:** CRGs were closely related to the immune pattern and self-renewal biology of cancer cells in MM. This prognostic model provided a new perspective for the risk stratification and treatment response prediction of MM patients.

## 1 Introduction

Multiple myeloma (MM) is a cytogenetically heterogeneous malignant plasma cell proliferative disorder and is the second most common hematologic malignancy (Laubach et al., 2011; Siegel et al., 2021). The clinical features, attributed to monoclonal protein, or cytokines secreted by the malignant plasma cells, are referred to as CRAB characteristics, including hypercalcemia, renal insufficiency, anemia, and osteolytic bone lesions (Rajkumar et al., 2014). MM may precede a precancerous state of MGUS and an asymptomatic stage called SMM (van de Donk et al., 2021).

The prognosis of MM patients has significantly improved over the past decade, mainly due to the application of novel therapies. However, survival outcome heterogeneity and relapse still persist in MM patients (Landgren and Rajkumar, 2016; Wallington-Beddoe et al., 2018). Therefore, more accurate methods are needed to better risk stratify MM patients for predicting survival and therapy decisions (Chng et al., 2014). The Revised International Staging System (R-ISS) is currently the most widely used prognostic staging system, incorporating the ISS with high-risk cytogenetic alterations [del (17p), t (4; 14) (p16; q32) or t (14; 16) (q32; q23)] and serum lactate dehydrogenase (LDH) (Palumbo et al., 2015). Whilst it has been validated in an analysis of an independent cohort of unselected patients with MM (Kastritis et al., 2017), it does not better reclassify patients from the ISS, suggesting that more reliable prognostic factors are needed to clarify the prognosis of patients (Cho et al., 2017; Jung et al., 2018).

Copper is one of the essential trace elements for all organisms (Kim et al., 2008). However, if the concentration exceeds a certain threshold, it can cause cell death. Recently, Tsvetkov et al. further clarified the potential mechanism of copper-induced toxicity and proposed a novel form of cell death termed cuproptosis (Tsvetkov et al., 2022). Depending on mitochondrial respiration, cuproptosis occurs through the direct combination of copper with the lipoylated components of the tricarboxylic acid (TCA) cycle. This leads to the aggregation of lipoylated proteins and the subsequent loss of iron-sulfur cluster protein, resulting in proteotoxic stress and eventually cell death (Tsvetkov et al., 2022).

Not only is the intrinsic apoptotic pathway most directly regulated by mitochondria (Carneiro and El-Deiry, 2020), but other forms of cell death, including autophagy (Gozuacik and Kimchi, 2004), necroptosis (Weinlich et al., 2017), pyroptosis (Bergsbaken et al., 2009), ferroptosis (Dixon et al., 2012) and, more recently, cuproptosis, are also tightly regulated by mitochondria. Mitochondria are the central hub of copper metabolism and homeostasis (Ruiz et al., 2021). Meanwhile, mitochondrial metabolism plays a determining role in the growth, survival, and therapeutic outcome of MM. MM is a malignancy of antibody-producing plasma cells from differentiated B cells. Increased expression of mitochondrial biogenesis and oxidative phosphorylation (OXPHOS) marker genes was consistently found in MM cells compared to normal plasma cells. Furthermore, the expression of mitochondrial biogenetic characteristic genes in recurrent and drug-resistant MM patients is higher than that in newly diagnosed patients, which has been proven to be related to the progression of MM (Zhan et al., 2017). Studies performed on MM show high anti-MM efficacy for both *in-vitro* and *in-vivo* models (Skrott et al., 2017; Xu et al., 2020), even in bortezomib -resistant cells (Salem et al., 2015; Chroma et al., 2022) and myeloma stem cells (Jin et al., 2018), when using copper ionophores. However, the effect of cuproptosis on the prognosis of MM patients remains largely unknown.

In the current study, we aimed to construct a risk-scoring model related to cuproptosis to predict the prognosis of MM and guide clinical treatment. The model was further validated in two independent external datasets and our clinical cohort. Finally, we explored the heterogeneity of biological functional status and tumor microenvironment ( ) among subgroups to reveal the underlying mechanisms.

## 2 Materials and methods

### 2.1 Data acquisition

The gene expression data and corresponding clinical information of multiple myeloma patients were attained from the Gene Expression Omnibus (GEO) database (http://www.ncbi.nlm.nih.gov/geo/), including GSE136337, GSE24080, GSE4204, and GSE6477. The gene expression profiles were log2 transformed and normalized between different arrays. The dataset GSE136337 was used as the training set because it has detailed clinicopathological information, including age, sex, albumin, β2-microglobulin, LDH, t [4; 14], t [14; 16], del [17p], ISS, R-ISS staging and survival data. The GSE24080 and GSE4204 datasets were used for validation. The dataset GSE6477 was applied in the analysis of the difference in CRGs expression profile between normal subjects and MGUS, SMM, and active MM. Ten cuproptosis-related genes (*FDX1, LIAS, LIPT1, DLD, DLAT, PDHA1, PDHB, MTF1, GLS,* and *CDKN2A*) were retrieved from the literature (Tsvetkov et al., 2022).

### 2.2 Gene interaction network and the landscape of genetic alterations

The correlation network of 10 CRGs was derived from the “circlize” R package. The interaction network for the overlapping prognostic CRGs was generated by the STRING database (version 11.5) (Szklarczyk et al., 2011).

Given the limited data on myeloma, to determine the somatic mutations of 10 CRGs, the single nucleotide variant (SNV) data of these CRGs in all cancers of The Cancer Genome Atlas (TCGA) (https://portal.gdc.cancer.gov/) were mined in Gene Set Cancer Analysis (GSCA) (http://bioinfo.life.hust.edu.cn/GSCA/#/) [28]. We also used cBioPortal for Cancer Genomics (http://www.cbioportal.org/) to analyze the frequency of gene mutation and corresponding mutation sites on the chromosome in hematologic malignancies.

### 2.3 Construction and validation of a prognostic cuproptosis-related gene signature

The GSE136337 dataset was used as the training dataset to establish the cuproptosis-related risk score. Univariable Cox regression analysis was performed to obtain CRGs associated with prognosis (*p* < 0.05). To minimize the risk of overfitting, the Least absolute shrinkage and selection operator (LASSO) Cox regression analysis was applied to determine the best weighting coefficient of CRGs using the R software package “glmnet” (Friedman et al., 2010). After 1000-fold cross-validation of the maximum likelihood estimate of the penalty, the minimum criterion was determined by the optimal value of the penalty parameter λ, and finally, a cuproptosis-related prognostic model was constructed. The GSE24080 and GSE4204 datasets were used for validation. The risk scores of the subjects were calculated according to the normalized expression level of each gene and its corresponding regression coefficients. Stratifying patients into high- and low-risk groups by median risk score with the “survival” and “survminer” R packages.

### 2.4 Comprehensive analyses of the prognostic model

The R software package “pRRophetic” was performed to evaluate the chemotherapeutic sensitivity between different groups. The weighted gene co-expression network analysis (WGCNA) was performed to explore the potential mechanisms associated with the prognostic model using the “WGCNA” R package (Langfelder and Horvath, 2008; 2012). First, we used the gene expression profile of the training set to calculate the median absolute deviation (MAD) of each gene, excluding the top 25% of the genes with the lowest MAD. Then we filtered out the optimal soft threshold to construct the scale-free co-expression network. In addition, we performed an association analysis between modules and clinical traits. The key genes most related to the risk score were identified by WGCNA analysis. Furthermore, we used the metascape online website (https://metascape.org/gp/index.html) to implement the Gene Ontology (GO) analysis about the key genes (*p*-value cutoff: 0.01).

The Kyoto Encyclopedia of Genes and Genomes (KEGG) pathways were employed to reveal the underlying basis of cuproptosis-related risk score. Enriched pathways in different cuproptosis-related risk score datasets were evaluated by Gene Set Enrichment Analysis (GSEA v4.2.2 software, http://software.broadinstitute.org/gsea/login.jsp). *p* < 0.05 and a false discovery rate q < 0.25 were considered indicative of statistical significance. Multiple GSEA were carried out with the R package “ggplot2”. Glycolysis-related genomes and TCA cycling-related genomes were downloaded from GSEA (http://www.gsea-msigdb.org/gsea/msigdb). The Gene Set Variation Analysis (GSVA) method implemented in the R package “GSVA” was used to calculate the enrichment score of each sample in the glycolytic gene set and TCA cycle gene set (Hanzelmann et al., 2013).

### 2.5 Characterization of the TME and immunotherapy responsiveness based on the cuproptosis-related model

To eliminate the effect of different algorithms, we used three algorithms to assess the immune infiltration level between different subgroups, including the single-sample gene set enrichment analysis (ssGSEA), the xCell (Aran, 2020) and ESTIMATE (Yoshihara et al., 2013). These algorithms use gene expression data and cytogenetic signatures to infer the level of infiltrating stromal cells and immune cells in tumor tissue. The microenvironment score in xCell and the ESTIMATE score in ESTIMATE are the sum of their respective immune and stromal scores. Additionally, the ESTIMATE algorithm converts the ESTIMATE score into a [0,1] range for tumor purity prediction. The more immune cells and stromal cells in the sample, the lower the tumor purity. Tumor immune dysfunction and exclusion (TIDE) was performed to identify potential factors of tumor immune escape (Jiang et al., 2018). Furthermore, the mRNA expression-based stemness index (mRNAsi) was used to compute the stemness index according to one-class logistic regression (OCLR)-based transcriptomic and epigenetic signatures (Malta et al., 2018). Ultimately, the T cell inflamed score (TIS) (Ayers et al., 2017) and immunophenotype score (IPS) (Charoentong et al., 2017) were used to evaluate the sensitivity to immune checkpoint blockade (ICB). The TIS score was calculated based on 18 marker genes with ssGSEA. Because of the lack of *HLA-DRB1* in the dataset, we used 17 genes.

### 2.6 External validation of cuproptosis-related mutations using an online database

We used the Cancer Cell Line Encyclopedia database (CCLE, https://portals.broadinstitute.org/ccle) to further validate the expression of cuproptosis-related genes highlighted by the risk score in MM.

### 2.7 Establishing a predictive nomogram

A nomogram for the combined model including age, the ISS phase and cuproptosis-related risk score was constructed using the “rms” package. A calibration curve was plotted for self-verification of the nomogram. R-ISS, cuproptosis-related risk scores, and the nomogram were compared with time-dependent receiver operating characteristic curves (time-ROC curves) for 1-,3- and 5-year survival using the “timeROC” R package (Blanche et al., 2013).

### 2.8 Cell lines and cell culture

LP-1, I9.2, and U266 cells were obtained from Fenghui Biotechnology Co., Ltd. (Hunan, China). Cells were cultured in RPMI-1640 medium (Gibco, Shanghai, China) supplemented with 10% fetal bovine serum (FBS), 0.1 mg/mL streptomycin, and 100 U/mL penicillin G and incubated at 37°C and 5% CO2 in a humidified atmosphere.

### 2.9 Patients

31 MM patients were enrolled in the study at the Department of Clinical Hematology of the First Affiliated Hospital of Wenzhou Medical University. Diagnoses were based on the 2014 International Myeloma Working Group (IMWG) criteria (Rajkumar et al., 2014). Meanwhile, normal bone marrow samples from 14 healthy donors were collected as controls for PCR on cell lines and patient samples. The distribution of clinical parameters and clinicopathological characteristics of the patients is shown in Table 1. The baseline characteristics of the MM and control groups were equally consistent in terms of gender and age (*p* > 0.05). Informed consent was obtained from the subjects for all collected samples. The Ethics Committee of the First Affiliated Hospital of Wenzhou Medical University approved the study, and all procedures were conducted in compliance with the Declaration of Helsinki.

**TABLE 1:** The clinical characteristics of the subjects included in this experiment.

Characteristics	Levels	MM (n = 31)	Normal (n = 14)	*p*
Sex	Female	20 (65%)	8 (57%)	0.637
	Male	11 (35%)	6 (43%)	-
Age	≤65 years	9 (29%)	5 (36%)	0.920
	>65 years	22 (71%)	9 (64%)	-
Isotype	IgG	16 (52%)	-	-
	IgA	8 (26%)	-	-
	IgD	1 (3%)	-	-
	Light chain	6 (19%)	-	-
Albumin	≥3.5 g/dL	12 (39%)	-	-
	<3.5 g/dL	19 (61%)	-	-
β2M	<3.5 mg/L	11 (35%)	-	-
	3.5–5.5 mg/L	7 (23%)	-	-
	≥5.5 mg/L	13 (42%)	-	-
LDH	≤250 U/L	24 (77%)	-	-
	>250 U/L	7 (23%)	-	-
Del (17p)	False	31 (100%)	-	-
	True	0 (0%)	-	-
IgH rearrangement	False	30 (97%)	-	-
	True	1 (3%)	-	-
Del (13q)	False	22 (71%)	-	-
	True	9 (29%)	-	-
Amp1q	False	21 (68%)	-	-
	True	10 (32%)	-	-
ISS	I	4 (13%)	-	-
	II	14 (45%)	-	-
	III	13 (42%)	-	-
R-ISS	I	4 (13%)	-	-
	II	25 (81%)	-	-
	III	2 (6%)	-	-
Myeloma cells	<10%	11 (35%)	-	-
	≥10%	20 (65%)	-	-
Calcium	≤2.65 mmol/L	30 (97%)	-	-
	>2.65 mmol/L	1 (3%)	-	-
Serum creatinine	<177 μmol/L	24 (77%)	-	-
	≥177 μmol/L	7 (23%)	-	-
Hb	≥85 g/L	19 (61%)	-	-
	<85 g/L	12 (39%)	-	-
Bone lesions	0	11 (36%)	-	-
	1–3	2 (6%)	-	-
	>3	18 (58%)	-	-

### 2.10 RNA extraction, reverse transcription, and quantitative real-time PCR

The total RNA was extracted from bone marrow samples by Righton DNA&RNA Blood and Tissue Kit (Righton Bio, Shanghai, China) according to the manufacturer’s instructions. Reverse transcription was performed with the cDNA synthesis kit (Vazyme, Nanjing, China) to generate cDNAs. Quantitative PCR was performed to detect the expression levels of CRGs by using Taq Pro universal SYBR qPCR Master Mix (Vazyme, Nanjing, China), β-ACTIN served as an internal control. Relative expression was calculated using the comparative threshold cycle (Ct) method (Livak and Schmittgen, 2001). A complete list of primers used was shown below:

DLD forward primer (FP): 5′-GAA​ATG​TCC​GAA​GTT​CGC​TTG​A-3′; DLD reverse primer (RP): 5′-TCA​GCT​TTC​GTA​GCA​GTG​ACT-3′; PDHA1 FP: 5′-TGG​TAG​CAT​CCC​GTA​ATT​TTG​C-3′; PDHA1 RP: 5′-ATT​CGG​CGT​ACA​GTC​TGC​ATC-3′; MTF1 FP: 5′-CAC​AGT​CCA​GAC​AAC​AAC​ATC​A-3′; MTF1 RP: 5′-GCA​CCA​GTC​CGT​TTT​TAT​CCA​C-3′; LIPT1 FP: 5′-TTG​CTA​AAG​AGC​CCT​TAC​CAA​G-3′; LIPT1 RP: 5′-TCA​TCC​GTT​GGG​TTT​ATT​AGG​TG-3′; β-ACTIN FP: 5′-TCA​AGA​TCA​TTG​CTC​CTC​CTG​AG-3′; β-ACTIN RP: 5′-ACA​TCT​GCT​GGA​AGG​TGG​ACA-3′.

### 2.11 Statistical analyses

SPSS software vision 24.0 (SPSS, Inc., Chicago, IL, United States), GraphPad Prism 9.0.0, and R software vision 4.1.1 (R Foundation for Statistical Computing, Vienna, Austria) were used for statistical analyses. For quantitative variables, Student’s t-test is used to analyze the differences between groups of normal distribution variables, and Wilcoxon test is used for skewed data. To compare the CRG expression differences of the four groups (normal, MGUS, SMM, and MM), one-way ANOVA was used, followed by the LSD multiple comparisons test. And chi-square test is used for difference analysis of categorical variables. If not specified above, *p*-value <0.05 was considered statistically significant, and all *p* values were two-tailed.

## 3 Results

### 3.1 Subject selection and baseline clinical covariates

**FIGURE 1:**
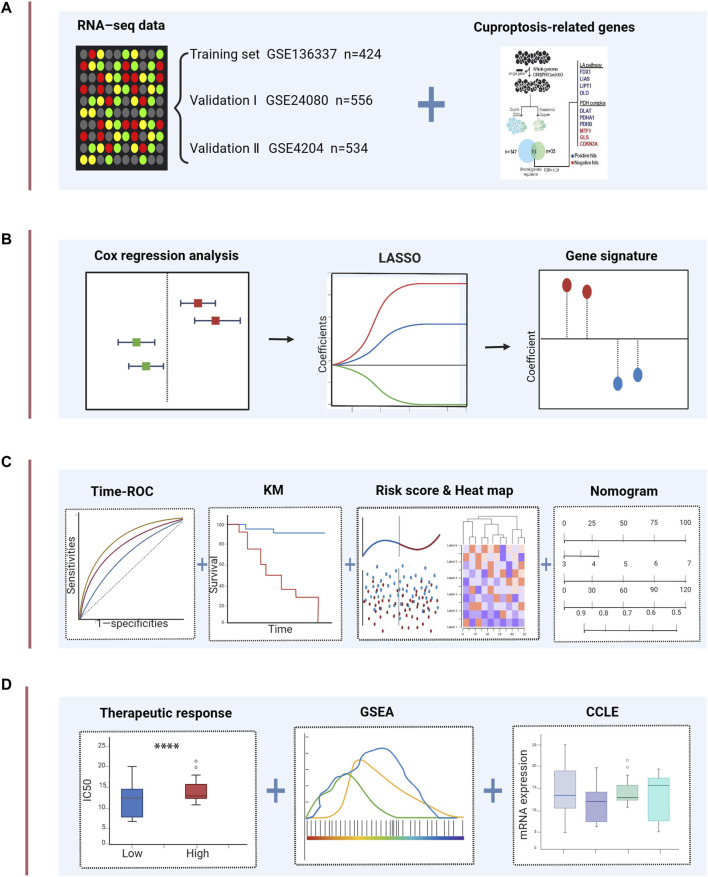
Schematic diagram of the study design. **(A)** The gene expression data and clinical information of MM patients were attained from the GEO database. 10 cuproptosis-related genes were retrieved from the literature. **(B)** Univariable Cox regression analysis and LASSO Cox algorithms were combined to develop the cuproptosis-related gene signature for prognosis. **(C)** The prognostic and predictive capacities of the model were validated in different datasets and methods. **(D)** Comprehensive analyses of therapeutic responses, and enriched pathways using GSEA and mRNA expression levels in CCLE. ****p < 0.0001.

The flow chart of this study is shown in Figure 1. The GSE136337 cohort was used to construct a cuproptosis-related prognostic risk score. The GSE24080 and GSE4204 datasets were used for model validation. Survival data were available for 1,514 subjects in the three datasets (GSE136337, n = 424; GSE24080, n = 556; GSE4204, n = 534). A sufficient number of subjects had clinical co-variates for univariate and multivariate Cox regression analysis in the training dataset (GSE136337; n = 415) and the first validation cohort (GSE24080; n = 556), but not in the second validation dataset (GSE4204). The detailed clinical characteristics of these patients are summarized in Table 2.

**TABLE 2:** Clinical covariates of the training and validation cohorts.

Characteristics	Training cohort	Validation cohort	Validation cohort
	GSE136337	GSE24080	GSE4204
	(n = 415)	(n = 556)	(n = 534)
Sex			
Female	158 (38%)	222 (40%)	-
Male	257 (62%)	334 (60%)	-
Age			
≤65 years	297 (72%)	421 (76%)	-
>65 years	118 (28%)	135 (24%)	-
Alb			
≥3.5 g/dL	331 (80%)	481 (87%)	-
<3.5 g/dL	84 (20%)	75 (13%)	-
β2M			
<3.5 mg/L	187 (45%)	320 (58%)	-
3.5–5.5 mg/L	109 (26%)	118 (21%)	-
≥5.5 mg/L	119 (29%)	118 (21%)	-
LDH			
≤250 U/L	392 (94%)	507 (91%)	-
>250 U/L	23 (6%)	49 (9%)	-
Del (17p)			
False	400 (96%)	-	-
True	15 (4%)	-	-
t (4,14)			
False	401 (97%)	-	-
True	14 (3%)	-	-
t (14,16)			
False	414 (99%)	-	-
True	1 (1%)	-	-
ISS			
I	163 (39%)	296 (53%)	-
II	133 (32%)	142 (26%)	-
III	119 (29%)	118 (21%)	-
R-ISS			
I	149 (36%)	-	-
II	243 (59%)	-	-
III	65 (16%)	-	-
Risk score			
High	209 (50%)	278 (50%)	267 (50%)
Low	206 (50%)	278 (50%)	267 (50%)
Survival			
Alive	239 (58%)	386 (69%)	442 (83%)

Alb albumin, β2M β2-microglobulin, LDH, lactate dehydrogenase.

### 3.2 Gene interaction networks and genetic alteration profiles of CRGs

**FIGURE 2:**
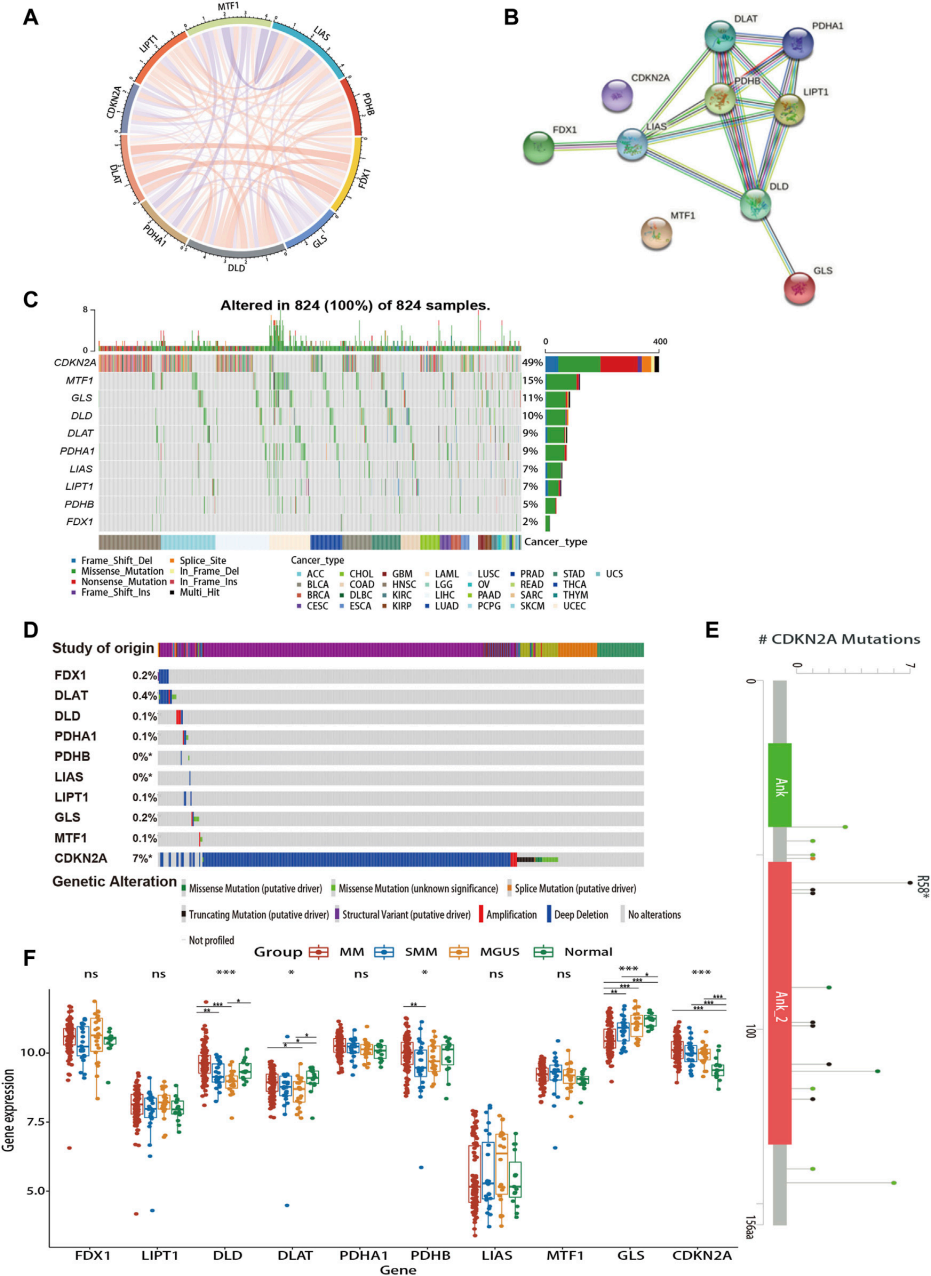
Gene interaction networks and genetic alteration profiles of CRGs. **(A)** The correlation network of candidate genes. The correlation coefficients are represented by different colors. **(B)** The PPI network downloaded from the STRING database indicated the interactions among the 10 candidate genes. **(C)** The genetic changes of CRGs in pan-cancer analysis **(D)** The genetic changes of CRGs in various hematologic malignancies. **(E)** Major mutation sites of *CDKN2A* in hematological malignancies. **(F)** Differences in CRG expression profile during the evolution of MM. PPI, protein-protein interaction; MGUS, monoclonal gammopathy of undetermined significance; SMM, smoldering myeloma. ns, no significance; *p < 0.05; **p < 0.01; ***p < 0.001.

As shown in Figures 2A,B, there is a close relationship between CRGs. Mutations in genes are closely associated with the occurrence and development of cancer. Due to the limited data on MM in the database, we explored the genetic alterations of CRGs in pan-cancer as well as hematologic tumors using TCGA and cBioPortal databases, respectively (Figures 2C,D). We found a wide range of mutations of CRGs in cancer. Among them, *CDKN2A* showed the highest mutation frequency both in two analyses. Figure 2E revealed the major mutation sites of *CDKN2A* in hematologic malignancies. In addition, to investigate the role of CRG expression profile in the development of MM, we further analyzed the differences in CRG expression between normal subjects, MGUS, SMM, and symptomatic MM with GSE6477 (Figure 2F). From normal group to SMM and MM, *GLS* showed a downward trend, and the expression of *DLD* in MM was higher than that of SMM and MGUS (*p* < 0.01). Similarly, the expression of *DLAT* in MM was higher than that of MGUS (*p* < 0.05). In MM, SMM and MGUS populations, the expression of *CDKN2A* was higher than that in the control group (*p* < 0.001).

### 3.3 Construction of a prognostic cuproptosis-related model

**FIGURE 3:**
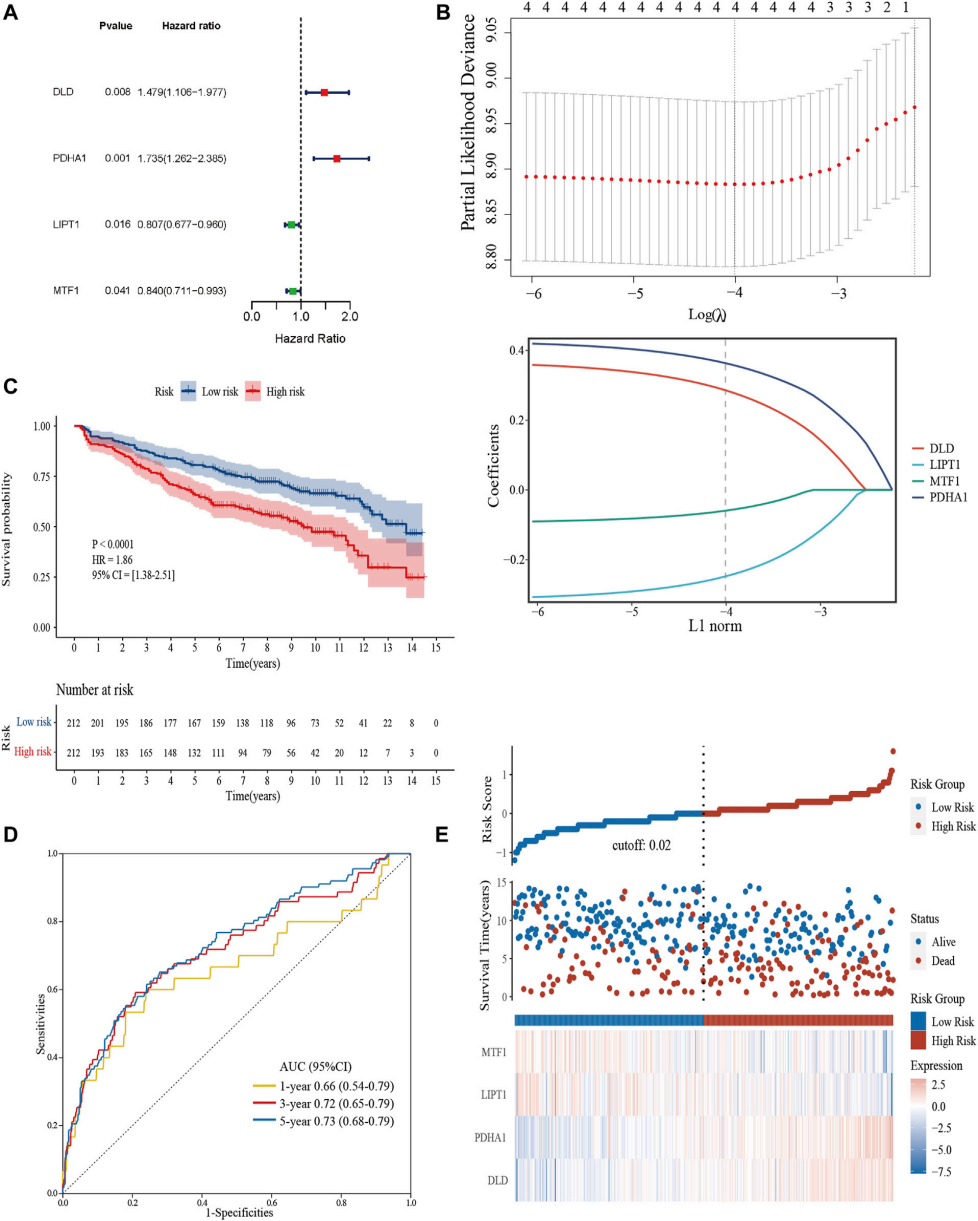
Construction and validation of the cuproptosis-related risk score model in the training cohort. **(A)** Forest plot of hazard ratios demonstrating the prognostic values of CRGs. **(B)** Construction of the prognostic model using LASSO. **(C)** Kaplan-Meier curves for the OS of subjects in the high-risk group and low-risk group. **(D)** The sensitivity and specificity of the cuproptosis-related risk score model were assessed by time-dependent ROC analysis. **(E)** Dot plots compared the survival outcomes of subjects between the high- and low-risk groups. The heat maps manifested the expressions of the four genes in the training cohort.

We extracted 10 candidate CRGs based on the literature (Tsvetkov et al., 2022). In the GSE136337 training dataset, 4 genes were significantly associated with survival in univariate Cox regression analysis (*p* < 0.05) (Figure 3A). Penalty maximum likelihood estimation was performed for 1,000 bootstrap replicates using lasso Cox regression analysis. The optimal weighting coefficient for each gene was determined by the regularization parameter lambda using the min standard. Four genes with high coefficients were selected to build the cuproptosis-related risk score (Figure 3B). The formula for the risk score was as follows: cuproptosis-related risk score = (0.2859× expression of *DLD*) + (0.3637× expression of *PDHA1*)—(0.2480× expression of *LIPT1*)—(0.0596× expression of *MTF1*). Using the established formula, a cuproptosis-related prognostic risk score for each sample was calculated. The patients were divided into high-risk and low-risk cohorts according to the median risk score of the corresponding datasets.

### 3.4 Validation of the prognostic cuproptosis-related risk score in each cohort

Kaplan–Meier curves were used to compare the survival of high- and low-risk groups in the training (Figure 3C) and validation datasets (Supplementary Figures S1A, S1D). Subjects in the high-risk group had worse survival compared to the low-risk group (HR = 1.86, 95% CI = 1.38–2.51, *p* < 0.0001; HR = 1.55, 95% CI = 1.14–2.10, *p* = 0.0048; HR = 1.53, 95% CI = 1.01–2.31, *p* = 0.043). To evaluate the prognostic accuracy of the model, time-dependent ROC analysis was conducted, with the AUC for the 1-, 3-, and 5-year survival being 0.66 (95% confidence interval [CI]: 0.54,0.79), 0.72 (0.65, 0.79), and 0.73 (0.68, 0.79) in the GSE136337 training dataset (Figure 3D). AUCs for the validation datasets are shown in Supplementary Figures S1C, S1D. Consistently, dot plots demonstrated that patients with higher risk scores exhibited worse overall survival in each dataset (Figure 3E). Moreover, the differences in the expression of four prognostic CRGs between high and low-risk groups were observed (Figure 3E).

### 3.5 Uni- and multi-variable analyses

To evaluate the independent prognostic force of the 4-gene signature, both the uni- and multi-variable Cox proportion hazard regression models were implemented (Table 3). We analyzed the clinicopathological traits correlated with survival, including sex, age, albumin, β2-microglobulin, LDH, t [4; 14], t [14; 16], del [17p], ISS and R-ISS phase in the GSE136337 training dataset, as well as sex, age, albumin, β2-microglobulin, LDH, and ISS stage in the first validation dataset (GSE24080). Results from the multi-variable analysis showed that the cuproptosis-related risk score was independently associated with survival with a HR = 1.658 (1.223, 2.246; *p* = 0.001) in the training dataset and HR = 1.437 (1.058, 1.952; *p* = 0.020) in the first validation dataset (GSE24080; Table 3).

**TABLE 3:** Univariate and multivariate Cox regression analyses of overall survival in the training and validation datasets.

Characteristics	Training cohort GSE136337 (n = 415)						Validation cohort GSE24080 (n = 556)				
	Univariate analysis			Multivariate analysis			Univariate analysis			Multivariate analysis	
	Regression coefficient (SE)	*p*		Hazard ratio (95% CI)	*p*		Regression coefficient (SE)	*p*		Hazard ratio (95% CI)	*p*
		
Age (<65 vs. ≥65 years)	0.579 (0.155)	<0.001		1.807 (1.33–2.455)	<0.001		0.174 (0.177)	0.327		-	-
Sex (female vs. male)	−0.248 (0.154)	0.107		-	-		−0.052 (0.156)	0.739		-	-
Albumin (≥3.5 vs. <3.5 g/dL)	0.410 (0.177)	0.021		-	-		0.595 (0.194)	0.002		-	-
β2m (<3.5 vs. 3.5–5.5 vs. ≥5.5 mg/L)	0.469 (0.091)	<0.001		-	-		0.512 (0.088)	<0.001		-	-
LDH (≤250 vs. >250 U/L)	0.732 (0.270)	0.007		-	-		1.316 (0.197)	<0.001		-	-
del (17p)	0.098 (0.417)	0.814		-	-		-	-		-	-
t (4,14)	0.035 (0.455)	0.939		-	-		-	-		-	-
t (14,16)	0.719 (1.003)	0.474		-	-		-	-		-	-
ISS (Ⅰ vs.Ⅱ vs.Ⅲ)	0.503 (0.095)	<0.001		1.584 (1.314–1.910)	<0.001		0.526 (0.090)	<0.001		1.659 (1.389–1.982)	<0.001
R−ISS (Ⅰ vs.Ⅱ vs.Ⅲ)	0.595 (0.133)	<0.001		-	-		-	-		-	-
Risk (low vs. high)	0.618 (0.155)	<0.001		1.658 (1.223–2.246)	0.001		0.435 (0.155)	0.005		1.437 (1.058–1.952)	0.020

Albumin, β2M, and LDH, were not included in the multivariate analysis, because of co-linearity with the ISS, or R-ISS.

Alb albumin, β2M β2-microglobulin, LDH, lactate dehydrogenase.

### 3.6 Comparative analysis between high- and low-risk groups

**FIGURE 4:**
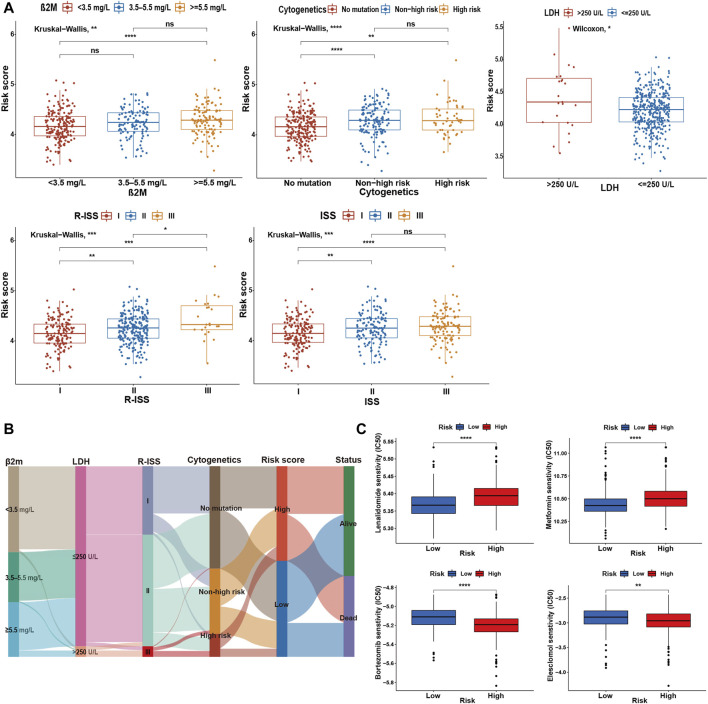
Comparative analysis between high- and low-risk groups. **(A)** Relationship between risk scores and clinicopathological parameters in GSE136337. **(B)** Sankey diagram showing the changes of β2M level, LDH level, R-ISS stage, cytogenetic mutation risk, cuproptosis-related risk score, and survival outcome. **(C)** Drug sensitivity assessment based on the cuproptosis-derived signatures in the training cohort. ns, no significance; *p < 0.05; **p < 0.01; ***p < 0.001; ****p < 0.0001.

To determine the specificity of the cuproptosis-related risk score in patients with different clinical features, we analyzed the relationship between clinical traits and risk scores in the GSE136337 training dataset. Patients with high β2M or LDH was found to be significantly associated with higher risk score (Figure 4A). With the increase of the ISS or R-ISS stage, the risk score also showed a gradually increasing trend (Figure 4A). Based on previous studies (Chng et al., 2014; Sonneveld et al., 2016; Abdallah et al., 2020; Wallington-Beddoe and Mynott, 2021), we defined high-risk cytogenetic abnormalities (HRCAs) as at least one of the following: del17p, amp1q, t (4; 14), t (14; 20), t (14; 16) or MYC aberrations determined by fluorescent *in situ* hybridization (FISH) or conventional karyotyping. Other abnormalities in the training set were classified as non-high-risk group [del13q, del16q, del1p, del1q, t (11; 14), t (12; 14)]. The rest were divided into the non-mutation group. A high score indicated a greater risk of cytogenetic mutations (Figure 4A). The sankey diagram was used to visualize the changes in patient characteristics (Figure 4B).

Further research indicated that the high-risk group was more resistant to lenalidomide and metformin, while it was more sensitive to bortezomib and elesclomol (a kind of copper ionophore), compared with the low-risk group (Figure 4C).

### 3.7 Characterization of TME and immunotherapy responsiveness based on the cuproptosis-related model

**FIGURE 5:**
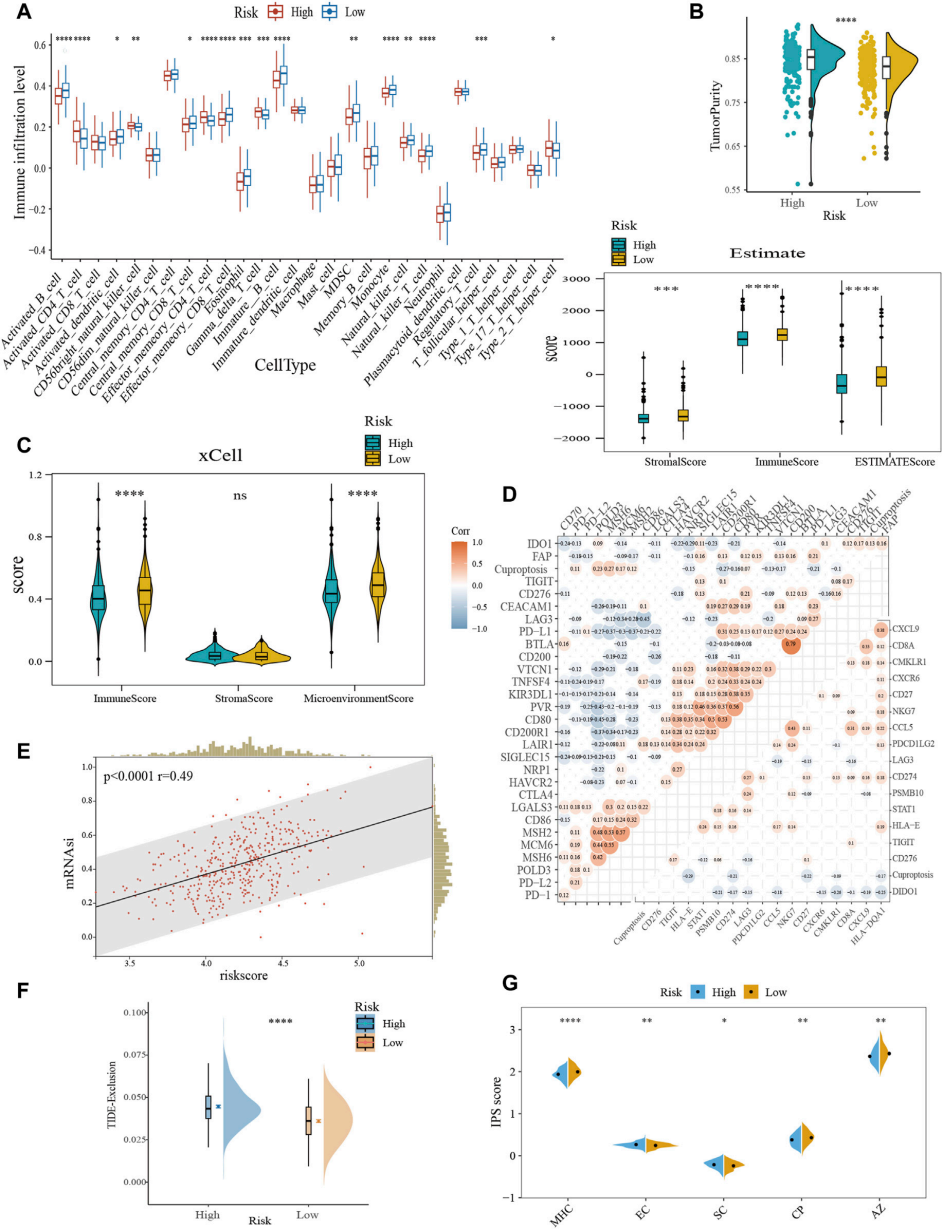
Characterization of TME and immunotherapy responsiveness based on the cuproptosis-related model. **(A)** Differences in immune infiltration between the cuproptosis subgroups with ssGSEA algorithm. **(B)** Evaluation of the immune infiltration with the ESTIMATE method. **(C)** Comparison of immune scores obtained from the xCell method. **(D)** Correlation analysis between risk score and immune checkpoint expression (upper left) and TIS (bottom right). **(E)** The association between the risk score and mRNAsi. **(F)** Differences in TIDE score between the cuproptosis subtypes. **(G)** Comparative analysis of IPS scores between the high- and low-scoring groups. TIS, T cell inflamed score; mRNAsi, mRNA expression-based stemness index; TIDE, tumor immune dysfunction and exclusion; IPS, immunophenotype score; MHC, antigen presentation; EC, effector cells; SC, suppressor cells; CP, checkpoint marker; z-score, AZ. ns, no significance; *p < 0.05; **p < 0.01; ***p < 0.001; ****p < 0.0001.

Overall, the proportion of immune cell infiltration was higher in the TME of the low-risk group, such as activated B cells, central memory CD8^+^ T cells, effector memory CD8^+^ T cells, NK cells, and activated dendritic cells (Figure 5A). Figures 5B,C further confirmed our findings. The low-risk group exhibited higher immune cell abundance, while the high-risk group possessed higher tumor purity. In addition, cuproptosis-related scores were negatively correlated with the expression levels of immune checkpoint and TIS gene signatures (Figure 5D). TIS can reflect sustained adaptive Th1 and cytotoxic CD8^+^ T cell responses. Having a high TIS implies high responsiveness to anti-PD-1/PD-L1 drugs (Ayers et al., 2017). mRNAsi was used to estimate the ability of cancer cells to self-renew. Correlation analysis demonstrated a positive correlation between risk score and mRNAsi (Figure 5E), implying a higher risk of recurrence in the high-scoring group. TIDE evaluates the level of tumor immune escape by assessing the dysfunction and exclusion of T cells. The higher the TIDE score, the higher the likelihood of immune escape, indicating that the patient is less responsive to ICB treatment. We found that the high-risk group was more likely to have a state that prevents T-cell infiltration (Figure 5F). IPS can visualize four different immunophenotypes (antigen-presenting, effector, suppressor cells, and checkpoint markers) in tumor samples (Charoentong et al., 2017). At the same time, it can generate a z-score with the combination of these four categories. The higher the z-score, the more immunogenic the tumor is and the more sensitive it is to immunotherapy. Consistently, the low-risk group had a higher IPS z-score (Figure 5G). In conclusion, the low-scoring group may be more sensitive to ICB.

### 3.8 Building a combined nomogram

**FIGURE 6:**
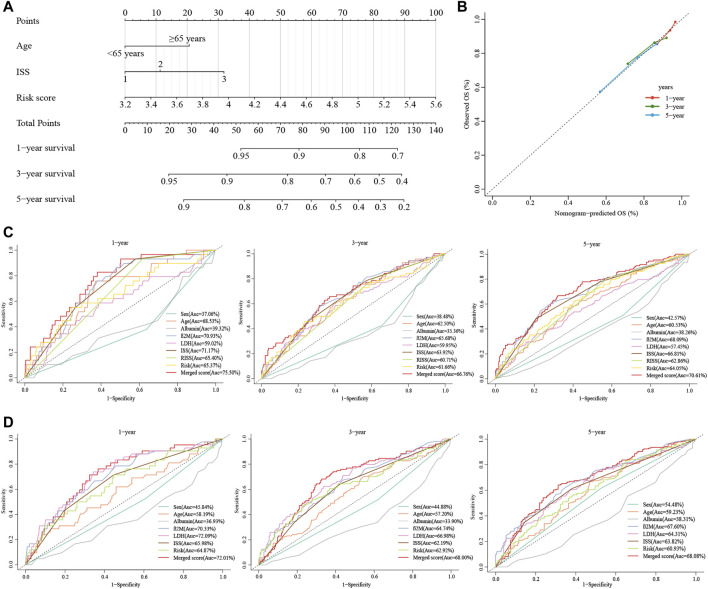
Construction of the nomogram for predicting survival in patients with MM. **(A)** The nomograms consisting of age, the ISS stage and risk score for predicting 1-, 3-, and 5-year OS. **(B)** Calibration curves of nomograms in terms of the agreement between predicted and observed 1-, 3-, and 5-year OS. **(C, D)** Time-dependent ROC curves for 1-, 3-, and 5-year OS predictions for the nomograms compared with other clinical covariates. C displays GSE136337, D displays GSE24080.

Due to the lack of cytogenetic data in the validation set, we constructed an integrated nomogram model featuring ISS stage, age, and cuproptosis-related risk score (Figure 6A). The calibration curve manifested a satisfactory agreement between predictive and observational values at the probabilities of 1-, 3- and 5-year survival (Figure 6B). The merged risk score showed more accuracy in predicting 1-, 3- and 5-year survival than other covariates including the ISS and R-ISS. In the training dataset, the nomogram improved the prediction accuracy from an AUC of 65.40% (0.59, 0.71), 60.71% (0.55, 0.67) and 62.86% (0.58, 0.68) of the R-ISS to an AUC of 75.50% (0.67, 0.84), 66.76% (0.60, 0.74) and 70.61% (0.65, 0.76) (Figure 6C). As for the GSE24080 validation dataset, the 1-, 3- and 5-year AUCs increased from 65.98% (0.58, 0.74), 62.19% (0.57, 0.68) and 63.82% (0.58, 0.69) of the ISS to an AUC of 72.01% (0.64, 0.80), 68.00% (0.62, 0.74) and 68.08% (0.62, 0.74) (Figure 6D).

### 3.9 Comprehensive analyses of biological function differences

**FIGURE 7:**
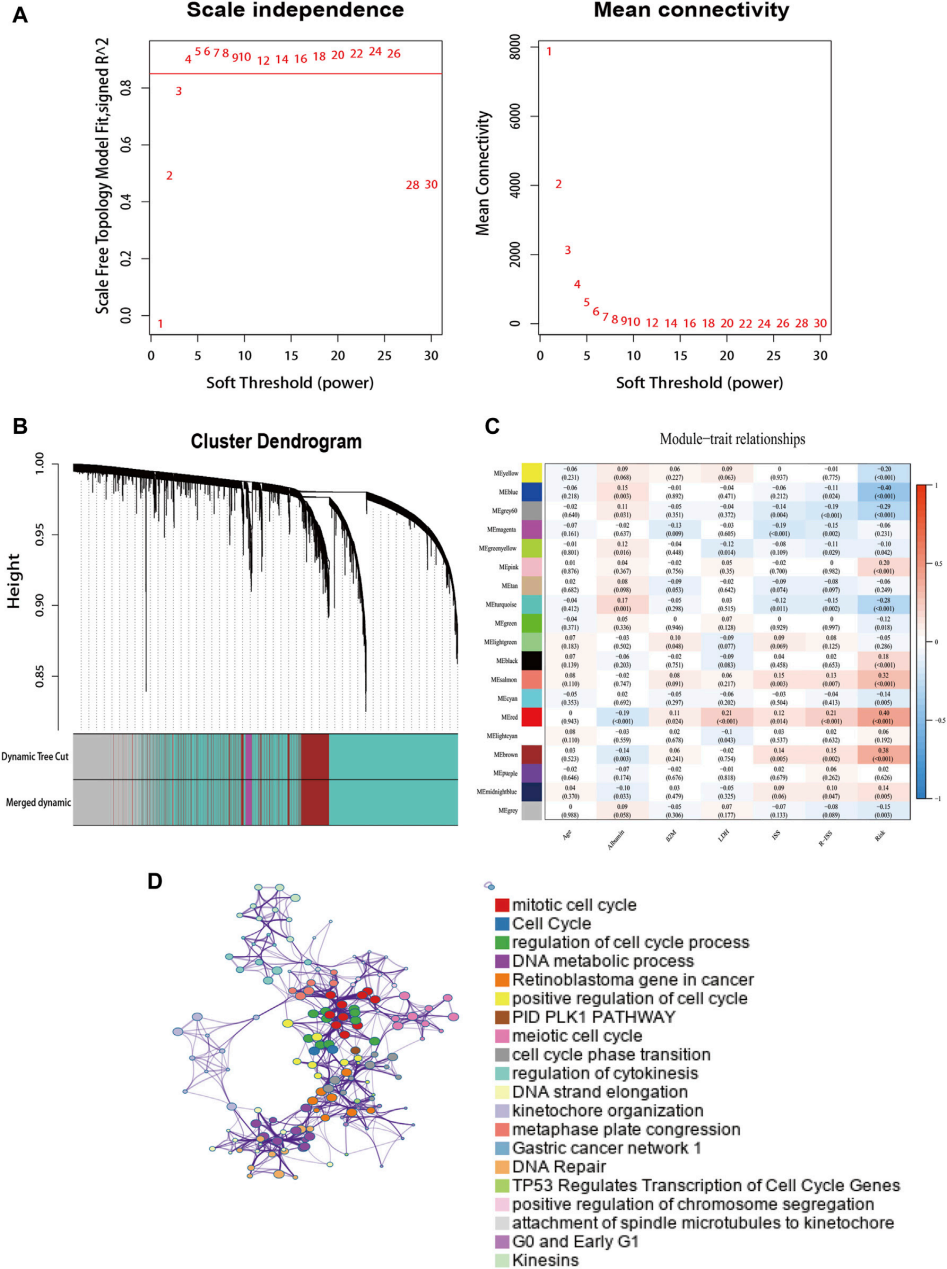
The weighted gene co-expression network. **(A)** Soft power value screening of genes in co-expression modules. **(B)** Hierarchical clustering dendrogram of genes. **(C)** Correlation analysis of gene module with risk model and clinical characteristics. **(D)** Enrichment clustering network in the Metascape database.

To determine the interaction of the risk model with other genes, we used WGCNA to construct a weighted gene co-expression network. The soft threshold parameter was set as four so that the threshold value of the adjacency matrix could meet the criterion of the network approaching scale-free (Figure 7A). These co-expression modules were then constructed and similar modules were clustered to finally obtain sixteen gene modules (Figure 7B). The results of the correlation analysis between gene modules and clinical characteristics showed that the red module had the highest correlation with risk scores (Correlation = 0.40, *p* < 0.001) (Figure 7C). The red module contains 284 genes, and then we performed GO functional enrichment analysis on these genes with Metascape and selected the top 20 clusters to construct a gene function clustering network (Figure 7D). Gene modules were mainly enriched in important biological processes of tumorigenesis and development. Such as mitotic cell cycle, DNA repair, and regulation of transcription of cell cycle genes by *TP53*.

**FIGURE 8:**
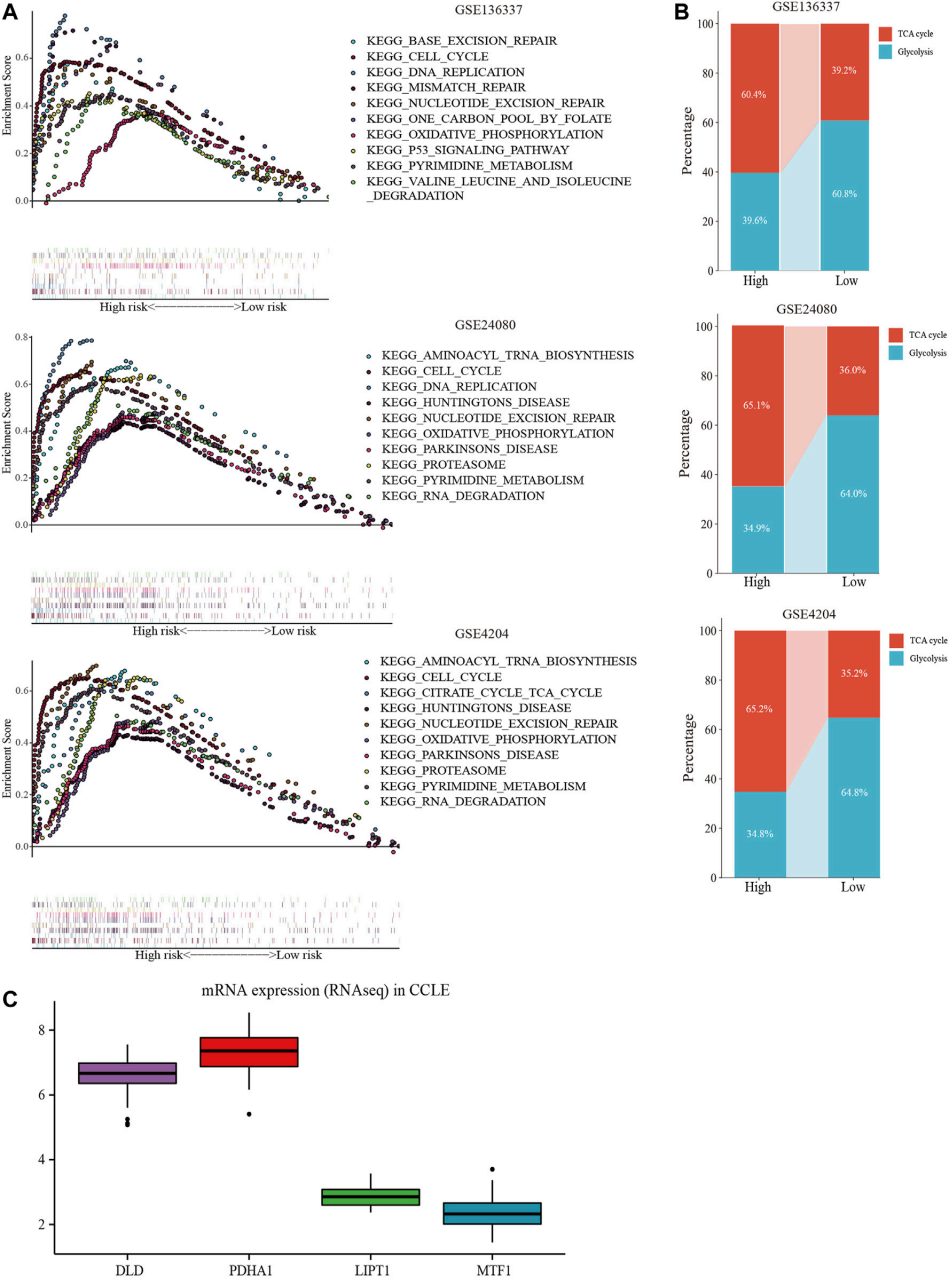
Comprehensive analyses of biological function differences. **(A)** The top 10 pathways were enriched in the training cohort and validation cohorts. **(B)** The different distribution ratios of glycolysis and TCA cycle. **(C)** mRNA expression by RNA sequencing of the four genes in the Cancer Cell Line Encyclopedia (CCLE) database.

GSEA was performed in each dataset to elucidate the biological functions and pathways that were associated with the risk score. Significantly enriched pathways were concentrated in the high-risk cohort and were mainly related to cuproptosis, including the TCA cycle, oxidative phosphorylation, valine leucine, and isoleucine degradation, pyrimidine metabolism, cell cycle, and DNA replication pathways (Figure 8A). Considering the close relationship between cuproptosis and the TCA cycle, we further used GSVA to calculate the per-sample overexpression level of the glycolytic gene list and TCA cycle gene set by comparing the ranks of the genes in that list with those of all other genes (Hanzelmann et al., 2013). The two groups exhibited different distribution ratios of glycolysis and TCA cycle. The TCA cycle was mainly concentrated in the high-risk group (Figure 8B).

In the CCLE database, *DLD* and *PDHA1* were over-expressed at the cellular level, while *LIPT1* and *MTF1* were under-expressed (Figure 8C), corresponding to the model equation above.

### 3.10 External experimental validation of prognostic signature

**FIGURE 9:**
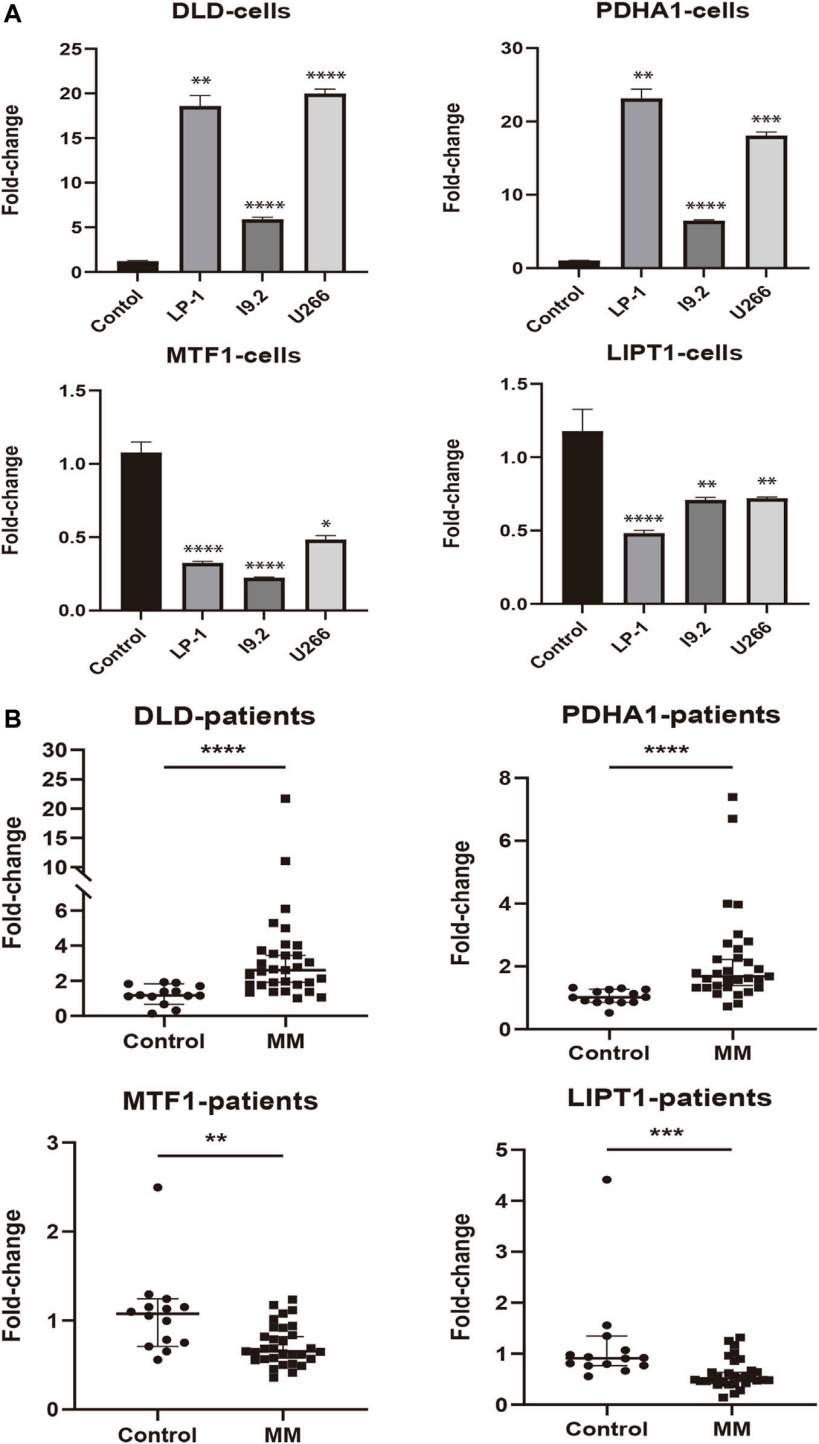
External experimental validation of prognostic signature. **(A)** Validation of prognostic CRGs expression in MM cell lines (LP-1, I9.2, and U266) (Mean ± SEM). **(B)** Expression of prognostic CRGs was compared by qRT-PCR in MM patients and control samples. *p < 0.05; **p < 0.01; ***p < 0.001; ****p < 0.0001.

To assess the expression status of these prognostic signature genes in MM, we first evaluated their expression in 3 MM cell lines (LP-1, I9.2, and U266) and the control group. As shown in Figure 9A, *DLD* and *PDHA1* were significantly upregulated in these 3 cell lines compared with the control group (*p* < 0.01). The opposite was true for *MTF1* and *LIPT1* (*p* < 0.05). We further examined the expression levels of prognostic CRGs in the bone marrow samples of 31 MM patients. Consistently, we found that the expression levels of *DLD* and *PDHA1* were significantly higher in MM samples than in healthy donors (*p* < 0.0001), and *MTF1* and *LIPT1* were significantly lower than in control samples (*p* < 0.01, Figure 9B).

## 4 Discussion

Changes in metabolism are increasingly recognized as one of the characteristics of cancer cells, including MM (Hanahan and Weinberg, 2011). Cancer cells depend on specific aspects of mitochondrial function for growth and survival, despite exhibiting elevated aerobic glycolysis (Weinhouse, 1956; Dalva-Aydemir et al., 2015; Porporato et al., 2018). Furthermore, mitochondria are involved in the regulation of multiple forms of cell death, such as apoptosis (Carneiro and El-Deiry, 2020), autophagy (Gozuacik and Kimchi, 2004), necroptosis (Weinlich et al., 2017), pyroptosis (Bergsbaken et al., 2009), ferroptosis (Dixon et al., 2012), and, more recently, cuproptosis (Tsvetkov et al., 2022). Cuproptosis, a recently proposed form of copper-dependent cell death, is mainly characterized by the proteotoxic stress induced by the direct combination of intracellular copper ions and lipoylated components in the TCA cycle (Tsvetkov et al., 2022).

MM is characterized by the accumulation of clonal plasma cells in the bone marrow and the consequently elevated immunoglobulins in serum and/or urine. And the differentiation of plasma cells begins with the activation of naive B cells, which leads to increased glucose uptake and promotes glycolysis and OXPHOS (Doughty et al., 2006). Activated B cells were observed to show progressive upregulation of the TCA cycle and electron transport chain (ETC.) genes to support increased OXPHOS and eventually immunoglobulin production (Price et al., 2018; Waters et al., 2018). In addition, MM cells exhibit addiction to glutamine (Bolzoni et al., 2016). And glutamine is the main carbon source for the TCA cycle in many cancer cells, including MM (DeBerardinis et al., 2007; Bajpai et al., 2016; Bolzoni et al., 2016). Mitochondrial transfer has been shown an important function in restoring mitochondrial respiration in cancer cells with damaged mitochondria and in assisting cells to escape apoptosis (Wang and Gerdes, 2015; Porporato et al., 2018). Similarly, it has been reported the mitochondrial exchange between stromal cells and MM cells in the myeloma microenvironment (Marlein et al., 2019). Translocated mitochondria were found to promote tumor progression and contribute to the survival response of cancer cells to chemotherapy (Marlein et al., 2019).

Therefore, the increase of OXPHOS is a critical plasma cell-specific metabolic dependence, and mitochondrial function plays an important role in maintaining plasma cell biology. Moreover, gene expression profiles associated with the mitochondrial TCA cycle and the, ETC predispose MM patients to bortezomib resistance and poor prognosis (Song et al., 2013; Tomlin et al., 2017; Zaal et al., 2022).

Given the important role of copper homeostasis and mitochondrial function in MM, targeted therapy of copper and the mitochondrial metabolism is a key and promising strategy in cancer therapy. Mitochondrial respiration complex I inhibitor metformin and glucose uptake inhibitor ritonavir could cooperatively induce cell death in MM cells (Dalva-Aydemir et al., 2015). Many previous studies have also revealed the great potential of Cu metal-binding compounds in cancer treatment. Among these, Cu ionophores can induce cuproptosis by increasing the intracellular Cu levels (Steinbrueck et al., 2020; Oliveri, 2022). Many different kinds of Cu ionophores have been used as anticancer agents to promote cuproptosis, including elesclomol (ES) and diethyldithiocarbamate (DTC), which is the active form of the disulfiram (DSF) (Denoyer et al., 2015; Hunsaker and Franz, 2019; Lelievre et al., 2020; Ge et al., 2022). ES has demonstrated its powerful targeted-killing effect on drug-resistant cancer cells, including cisplatin and proteasome inhibitor resistance (Wangpaichitr et al., 2009). Moreover, in both solid and hematological tumors, Cu-DSF has been shown to preferentially target cancer cells and can also selectively target and kill ALDH^+^ MM stem cell populations that lead to chemoradiotherapy resistance and recurrence (Yip et al., 2011; Liu et al., 2013; Xu et al., 2017; Jin et al., 2018; Wu et al., 2019; Sun et al., 2020). Studies have shown the potent anti-myeloma activity of Cu-DSF both *in vivo* and *in vitro*, even in cells resistant to proteasome inhibitors (Salem et al., 2015; Skrott et al., 2017; Xu et al., 2020). Cu-DSF kills MM cells, independent of disease stage and treatment (Chroma et al., 2022). Recently, a phase I, open-label trial of disulfiram in combination with copper gluconate in patients with treatment-refractory MM is underway (NCT04521335). However, studies investigating cuproptosis-related genes and their prognostic value in MM patients are limited.

The R-ISS is now the most widely accepted as standard prognostic model for MM patients. However, relevant studies have shown that stage II included populations with more heterogeneous survival outcomes (Cho et al., 2017; Jung et al., 2018). Many biomarkers that may improve the prediction accuracy of R-ISS have been studied, such as circulating tumor cells (CTCs) (Gonsalves et al., 2014; Chakraborty et al., 2016), 18F-fludeoxyglucose positron emission/computed tomography (Fonti et al., 2012; Zamagni et al., 2015), bone turnover markers (Patel et al., 2014), serum free light chain (FLC) levels (El Naggar et al., 2015) and genomics including next-generation sequencing (Bolli et al., 2020). But these indicators still need to be further verified. Therefore, the purpose of this study was to explore the cuproptosis-related prognostic biomarkers of MM, hoping to provide a new perspective on the risk stratification of MM patients.

In the current study, we constructed a novel prognostic model integrating four cuproptosis-related genes and validated it in two independent external cohorts. In the Cox regression analysis, the risk score was identified as an independent prognostic factor. Patients with different risk scores showed significantly different clinicopathological characteristics and drug susceptibility. Compared with the low-risk group, the high-risk group was confirmed to have higher levels of β2m and LDH, and higher R-ISS or ISS stages, as well as the probability of cytogenetic abnormalities. As for drug sensitivity, the high-risk group showed more sensitivity to bortezomib and ES, while it was resistant to lenalidomide and metformin, which is an inhibitor of mitochondrial complex Ⅰ. ES is a copper ionophore compound. In the previous research, ES demonstrated synergistic enhancement of bortezomib in the *in situ* mouse model of MM (Tsvetkov et al., 2019). The combination of ES and bortezomib or other proteasome inhibitors may provide a viable therapeutic strategy for MM.

Additionally, we found that cuproptosis-related scores could be applied to predict the level of immune infiltration, the ability of cancer cells to self-renew and differentiate in the TME of MM patients, and the sensitivity to ICB treatment. The low-risk group had a higher abundance of immune cells and, correspondingly, a higher sensitivity to immunotherapy. Moreover, the risk score was observed a positive correlation with the stemness index. Activation of cancer stem cells has been considered as the critical driving factor of tumor metastasis, recurrence, progression, and drug resistance (Clarke, 2019; Xu et al., 2021).

In the consensus on risk stratification, the IMWG has proposed that prognostic markers can be divided into host factors, tumor-related factors, tumor burden/stage, and treatment responsiveness, among which age is the most important host factor, while genetic aberration and gene expression profile are the most important tumor factors (Chng et al., 2014). Most previous studies on prognostic models of MM, including those investigating ISS, R-ISS, and gene expression profile (GEP), have concentrated only on tumor-related prognostic factors (Greipp et al., 2005; Kuiper et al., 2012; Kuiper et al., 2015; Palumbo et al., 2015). However, integrating tumor-related and patient-related factors should also be considered as an important strategy for the improvement of the staging system. Thus, in order to improve the ability for survival prediction, we established a nomogram combined with the ISS, age, and cuproptosis-related risk score to quantify the risk assessment. Compared to other traditional features including the R-ISS, the nomogram exhibited the highest accuracy and discrimination in survival prediction.

As mentioned above, mitochondrial respiration plays an important role in MM. Meanwhile, copper induces cell death by targeting lipoylated TCA cycle proteins, mitochondrial respiration is required for copper-induced cell death (Tsvetkov et al., 2022). The GSEA displayed that TCA cycle-related pathways were highly enriched in the high-risk score group. The TCA cycle is the ultimate metabolic pathway for sugars, lipids, and amino acids. The degradation of valine, leucine, and isoleucine provides the feedstock for the TCA cycle, the intermediate products of which are involved in pyrimidine synthesis and oxidative phosphorylation for the synthesis of ATP. Correspondingly, the GSVA also showed a higher proportion of TCA in the high-risk group than that in the low-risk group. In conclusion, the high-scoring group was more closely related to the TCA cycle. Moreover, we further verified the abnormal expression of these four genes through the online database CCLE and our *in vitro* experiments. The gene expression trends were consistent with our results.

In our prognostic model, *DLD* and *PDHA1* were shown to be risk-associated genes, whereas *LIPT1* and *MTF1* were identified as protective genes. The pyruvate dehydrogenase (PDH) complex is a nuclear-encoded mitochondrial multienzyme complex that catalyzes the overall conversion of pyruvate to acetyl-CoA and CO_2_ and provides the primary link between glycolysis and the TCA cycle. The PDH complex is composed of pyruvate dehydrogenase (PDH, E1), dihydrolipoamide acetyltransferase (*DLAT*, E2), and lipoamide dehydrogenase (*DLD,* E3) (Yu et al., 2008). *PDHA1*, the main regulatory site of PDH activity, is considered to promote cuproptosis (Dahl et al., 1992; Tsvetkov et al., 2022). PDH is a potential therapeutic target for MM. *PDHA* was found to enhance the anti-MM effect of bortezomib by modulating metabolic reorganization (Findlay et al., 2023). Furthermore, pyruvate dehydrogenase kinase 1 (PDK1) is a PDH inactivator. Studies have shown that PDK1 inhibitors such as JX06 and dichloroacetate can induce cell cycle arrest, apoptosis, and can synergistically kill MM cells with bortezomib (Fujiwara et al., 2013; Sanchez et al., 2013; Kawano et al., 2022). The downregulation of *DLD* expression was shown to increase intracellular ROS production and reduce mitochondrial membrane potential, thereby inducing autophagic cell death in melanoma cells and significantly inhibiting tumor proliferation *in vivo* (Yumnam et al., 2021). However, other studies have also noted that human cancer cells express *DLD* at lower levels than normal cells, which is associated with poor survival outcomes in several cancers, including kidney, colon, and cervical cancers (Shin et al., 2020). Recent studies show that *DLD* and *PDHA* have transcriptomic alterations in the progression of MM, which are associated with poor prognosis (Findlay et al., 2023). *LIPT1,* an enzyme that activates 2-ketoacid dehydrogenases related to the TCA cycle and promotes cuproptosis (Solmonson and DeBerardinis, 2018), has been reported the association with the prognosis of urothelial carcinoma and lung cancer in the Pathology Atlas project (Uhlen et al., 2017). *MTF1* protects cells from heavy metals by binding toxic metal ions to activate metallothionein expression (Takahashi, 2015). Increased expression of *MTF1* could lead to cell cycle arrest and apoptotic responses of B-cell lymphoma lines (Lecane et al., 2005). Additionally, its deletion would enhance the tolerance of chronic myeloid leukemia to arsenic trioxide (Sobh et al., 2019). However, another research has also found that *MTF1* shows high expression levels in glioma cells, and its knockout inhibits malignant progression (Ruan et al., 2020). Whether these genes play a role in the prognosis of MM patients by affecting the process of cuproptosis remains to be elucidated, as few relevant studies have been reported on these genes, especially in MM and even hematological tumors.

Our study, however, had several limitations that should be addressed. First, our prognostic model was constructed and validated with retrospective data from public databases and our clinical samples, so the prognostic robustness and clinical usefulness of the CRG signature need further validation in larger prospective studies. Second, the validation datasets we used lacked complete clinical information, such as R-ISS. Finally, the potential complex molecular mechanisms of CRGs in MM and the prognostic and diagnostic value of each gene still need to be explored and validated in more experiments and large sample clinical trials.

## 5 Conclusion

Overall, this study provides new insight into understanding the relationship between cuproptosis and MM. The prognostic model related to cuproptosis characterized the heterogeneity of clinicopathological features, treatment responsiveness, TME, and prognosis in MM patients. These findings may provide a feasible strategy for predicting clinical outcomes in MM, individualized treatment based on risk scores and developing new therapeutic targets.

## Figures and Tables

**Table:** 

**MM**	Multiple myeloma
**MGUS**	monoclonal gammopathy of undetermined significance
**SMM**	smoldering myeloma
**CRGs**	cuproptosis-related genes
**qRT-PCR**	quantitative real-time PCR
**TME**	tumor microenvironment
**R-ISS**	The Revised International Staging System
**HRCAs**	high-risk cytogenetic alterations
**LDH**	lactate dehydrogenase
**β2M**	β2-microglobulin
**TCA cycle**	tricarboxylic acid cycle
**OXPHOS**	oxidative phosphorylation
**RNA-seq**	RNA sequencing
**GEO**	the Gene Expression Omnibus
**SNV**	single nucleotide variant
**TCGA**	The Cancer Genome Atlas
**GSCA**	Gene Set Cancer Analysis
**LASSO**	the Least absolute shrinkage and selection operator
**WGCNA**	the weighted gene co-expression network analysis
**MAD**	median absolute deviation
**GO**	the Gene Ontology
**KEGG**	the Kyoto Encyclopedia of Genes and Genomes
**GSEA**	Gene Set Enrichment Analysis
**GSVA**	the Gene Set Variation Analysis
**ssGSEA**	single-sample gene set enrichment analysis
**TIDE**	tumor immune dysfunction and exclusion
**mRNAsi**	mRNA expression-based stemness index
**OCLR**	one-class logistic regression
**TIS**	T cell inflamed score
**IPS**	immunophenotype score
**MHC**	antigen presentation
**EC**	effector cells
**SC**	suppressor cells
**CP**	checkpoint markers
**ICP**	immune checkpoint blockade
**CCLE**	the Cancer Cell Line Encyclopedia database
**time-ROC curves**	time-dependent receiver operating characteristic curves
**IMWG**	International Myeloma Working Group
**FP**	forward primer
**RP**	reverse primer
**PPI**	protein-protein interaction
**FISH**	fluorescent *in situ* hybridization
**ETC**	electron transport chain
**ES**	elesclomol
**DTC**	diethyldithiocarbamate
**DSF**	disulfiram
**CTCs**	circulating tumor cells
**FLC**	free light chain
**GEP**	gene expression profile

## Data Availability

The datasets presented in this study can be found in online repositories. The names of the repository/repositories and accession number(s) can be found in the article/Supplementary Material.
