# Supplementary figures and images for: Identification and validation of a novel cuproptosis-related gene signature in multiple myeloma
Source: Front Cell Dev Biol. 2023 Apr 20;11:1159355. doi: 10.3389/fcell.2023.1159355 (PMC10157051; doi:10.3389/fcell.2023.1159355)

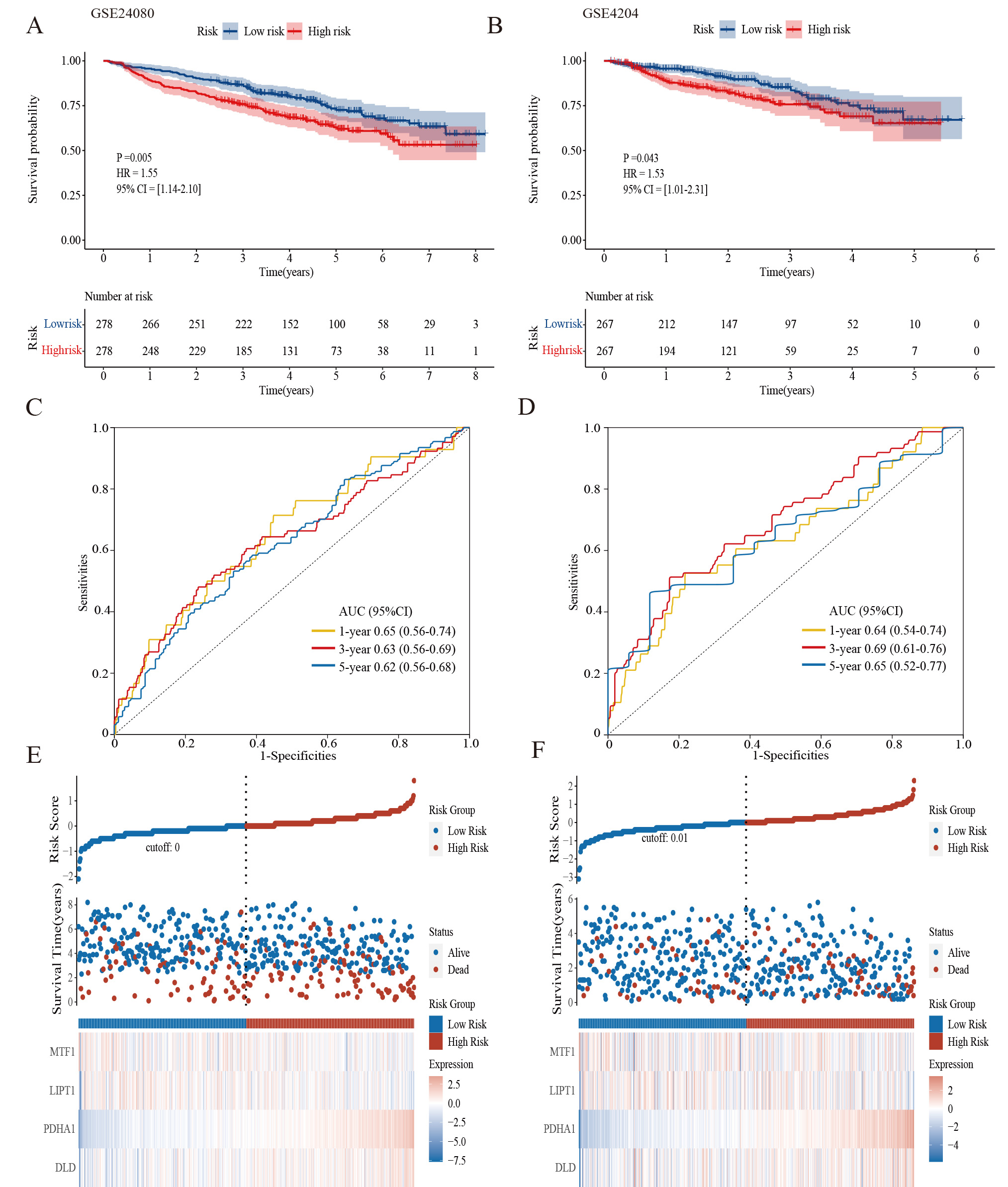

Supplement: Supplementary file 1 [file Image1.JPEG]
